# Giving rheumatology patients online home access to their electronic medical record (EMR): advantages, drawbacks and preconditions according to care providers

**DOI:** 10.1007/s00296-012-2408-2

**Published:** 2012-03-28

**Authors:** Rosalie van der Vaart, Constance H. C. Drossaert, Erik Taal, Mart A. F. J. van de Laar

**Affiliations:** 1 Department of Psychology, Health & Technology, University of Twente, Citadel H423, P. O. Box 217, 7500 AE Enschede, The Netherlands; 2 Arthritis Centre Twente, Medisch Spectrum Twente, Enschede, The Netherlands

**Keywords:** Electronic medical record, Patient access, Patient portal, Empowerment, Quality of care, eHealth

## Abstract

Technology enables patients home access to their electronic medical record (EMR), via a patient portal. This study aims to analyse (dis)advantages, preconditions and suitable content for this service, according to rheumatology health professionals. A two-phase policy Delphi study was conducted. First, interviews were performed with nurses/nurse practitioners (*n* = 9) and rheumatologists (*n* = 13). Subsequently, collected responses were quantified, using a questionnaire among the interviewees. The following advantages of patient home access to the EMR were reported: (1) enhancement of patient participation in treatment, (2) increased knowledge and self-management, (3) improved patient–provider interaction, (4) increased patient safety, and (5) better communication with others. Foreseen disadvantages of the service included: (1) problems with interpretation of data, (2) extra workload, (3) a change in consultation content, and (4) disturbing the patient–provider interaction. Also, the following preconditions emerged from the data: (1) optimal security, (2) no extra record, but a patient-accessible section, (3) no access to clinical notes, and (4) a lag time on the release of lab data. Most respondents reported that data on diagnosis, medication, treatment plan and consultations could be released to patients. On releasing more complex data, such as bodily examinations, lab results and radiological images the opinions differed considerably. Providing patients home access to their medical record might be a valuable next step into patient empowerment and in service towards the patient, provided that security is optimal and content and presentation of data are carefully considered.

## Introduction

Providing patients online home access to their electronic medical record (EMR) offers a new perspective on patient empowerment [1]. In several studies, including studies in rheumatology, patients are found to be eager to access their EMR, independent of age, race or education level [2–7]. Patients report that it would enhance involvement in their treatment and that it would give them the feeling of ownership of their own medical information [7]. However, despite potential benefits and patients’ positive attitudes, studies among health professionals in several areas show that professionals are more cautious towards providing patients home access to their EMR [2, 8, 9]. While health professionals have acknowledged benefits, such as increased patient knowledge and empowerment, or improved doctor–patient communication, many concerns about confused patients and increased workload still remain [2, 10]. As a result, the service of providing patients home access to their medical data remains scarcely implemented [9, 11].

In the field of rheumatology, patient home access to the EMR seems particularly useful, since patient self-management and patient empowerment are considered highly important. Moreover, rheumatology care providers often have a long-term treatment relationship with their patients, in which cooperation plays an important role [12]. Yet, no previous studies on providing rheumatology patients online access to their EMR’s have been published. Knowledge on how rheumatology care providers feel about offering their patients home access to their EMR is, therefore, lacking. The aim of this study was to analyse rheumatology care providers’ foreseen advantages and disadvantages towards this service, and to examine how this service could be used in clinical practice.

## Methods

A policy Delphi method was used [13], in which care providers’ opinions on patient home access to the EMR were explored, using qualitative interviews, and in which areas of (dis)agreement were identified in a quantitative survey containing questions based on the interview data [14, 15].

### Recruitment of participants

Twenty-two rheumatologists and thirteen nurses/nurse practitioners, from twelve hospitals spread over the Netherlands, were randomly selected from the most recent versions of the Dutch Rheumatology Association’s members lists [16, 17]. Thirteen rheumatologists (59 %) and nine nurses/nurse practitioners (69 %) from nine different hospitals agreed to participate in an interview. The interviews were scheduled at the care providers’ hospital and took approximately 45 min. Data saturation was reached, meaning that no new information of added value was obtained in the final three interviews [18].

### Phase 1: interviews

Each interview started off by asking care providers about their work-related Internet use, and their views on using a patient portal in rheumatologic care. The interviews continued by showing care providers a paper prototype of an online patient-accessible EMR, offering patients home access to their diagnosis, medication, treatment plan and latest lab results, and access to care providers’ clinical notes. The interview scheme contained the following fixed questions: “What would be the advantages/disadvantages of this service?” “Would the advantages/disadvantages differ for patients and care providers?” and “What preconditions or guidelines would you set for this service on a hospital based patient portal?”.

The audiotapes of the interviews were transcribed verbatim. Using content analysis, two independent researchers (RvdV and CHCD) selected quotes on advantages, disadvantages and preconditions, and coded them into categories. Subsequently, these categories were discussed between the two researchers, until consensus about the final categories was reached.

### Phase 2: questionnaire

In phase two, the categories of responses from phase 1 were translated into a questionnaire with pre-formulated answering categories. Each item could be answered on a 5-point Likert scale, ranging from “definitely yes” to “definitely no”. Tables 1 and 2 provide an overview of the categories and items on, respectively, advantages and disadvantages. Table 3 shows three additional items which reflect preconditions mentioned by the care providers in the interviews. Additionally, items were designed to examine suitable content for a patient home-accessible EMR (see Table 4). Results from the questionnaire were analysed by quantifying scores on each item and calculating percentages of care providers who agreed, or were neutral or disagreed on the items.

**Table 1:** Advantages of patient access to the EMR (arranged in categories obtained from phase 1) (*n* = 17)

Questionnaire item	Definitely/probably %^a^ (*n*)	Neutral %^a^ (*n*)	Definitely not/probably not %^a^ (*n*)
*Patient participation*			
It could help the patient prepare for consult	88 (15)	12 (2)	0
It could enhance the patient’s feeling of grip/control on the treatment	82 (14)	18 (3)	0
The patient could get more involved in treatment	82 (14)	18 (3)	0
It could enhance shared decision making	82 (14)	18 (3)	0
It could enhance autonomy/emancipation of the patient	65 (11)	29 (5)	6 (1)
*Knowledge & self*-*management*			
It could enhance insight/understanding in the disease	82 (14)	18 (3)	0
The patient could be better informed (*n* = 16)	81 (13)	19 (3)	0
It could enhance therapy adherence	77 (13)	18 (3)	6 (1)
*Patient: provider interaction*			
It could improve the trust that patients have in their provider	53 (9)	29 (5)	18 (3)
It could equalize patient–provider communication	47 (8)	41 (7)	12 (2)
It could enhance the patient–provider relationship	47 (8)	29 (5)	24 (4)
*Patient safety*			
It could enhance safety of care, for patients can read along and detect mistakes	59 (10)	24 (4)	18 (3)
*Communication with others*			
It could support patients in communicating with friends and family about their disease and treatment, because they can look at the record together	76 (13)	18 (3)	6 (1)
It is practical that the patient can take the record along to another hospital/GP/on vacation	76 (13)	18 (3)	6 (1)

^a^Percentages do not always add up to 100 % because of rounding

**Table 2:** Disadvantages of patient access to the EMR (arranged in categories obtained from phase 1) (*n* = 17)

Questionnaire item	Definitely/probably %^a^ (*n*)	Neutral %^a^ (*n*)	Definitely not/probably not %^a^ (*n*)
*Interpretation of data*			
Patients lack knowledge/insight for a good interpretation of the whole record	88 (15)	12 (2)	0
It could cause fear, stress or agitation	65 (11)	29 (5)	6 (1)
*Requires extra work/change in administration*			
As a care provider I will have to explain a lot more to patients	65 (11)	18 (3)	18 (3)
It will be difficult to (have to) write things differently (in layman’s terms)	47 (8)	18 (3)	35 (6)
*Change in consult*			
It could prevent me from being complete in my clinical notes (e.g. less personal interpretation)	53 (9)	24 (4)	24 (4)
The emphasis of the consult could lie too strongly on the EMR	29 (5)	53 (9)	18 (3)
*Patient: provider interaction*			
It could cause friction or a feeling of insult among patients (e.g. when they read care providers’ clinical notes)	53 (9)	41 (7)	6 (1)

^a^Percentages do not always add up to 100 % because of rounding

**Table 3:** Preconditions of patient access to the EMR (*n* = 17)

Questionnaire item	Definitely/probably %^a^ (*n*)	Neutral %^a^ (*n*)	Definitely not/probably not %^a^ (*n*)
There should be a separate section in the EMR which is not visible for patients	82 (14)	6 (1)	12 (2)
There should be a lag time on the release of lab results until after the consult	65 (11)	18 (3)	18 (3)
A patient-accessible EMR should be a copy of the existing EMR, and not an extra record	94 (16)	6 (1)	0

^a^Percentages do not always add up to 100 % because of rounding

**Table 4:** Content of the EMR that should be available for patients, according to care providers (*n* = 17)

Content	Positive %^a^ (*n*)	Neutral %^a^ (*n*)	Negative %^a^ (*n*)
*Basic information*			
Diagnosis	100 (17)	–	–
Allergies	100 (17)	–	–
Medical history	100 (17)	–	–
*Medication*			
Prescribed medication	100 (17)	0	0
(side)Effects medication	88 (15)	12 (2)	0
Medication history	82 (14)	18 (3)	0
Contraindications medication	59 (10)	35 (6)	6 (1)
*Consult information*			
Reason consult	88 (15)	6 (1)	6 (1)
Conclusion consult (*n* = 16)	88 (14)	–	13 (2)
Results anamnesis	53 (9)	29 (5)	18 (3)
Results physical examination	53 (9)	29 (5)	18 (3)
Clinical notes	18 (3)	12 (2)	71 (12)
Planned interventions	88 (15)	12 (2)	–
Treatment plan (*n* = 16)	100 (16)	–	–
Correspondence with GP	65 (11)	29 (5)	6 (1)
*Lab results*			
ESR value	82 (14)	18 (3)	–
CRP value	82 (14)	12 (2)	6 (1)
Liver function	65 (11)	24 (4)	12 (2)
Kidney function	59 (10)	29 (5)	12 (2)
Hb value	65 (11)	24 (4)	12 (2)
Full overview lab results	35 (6)	24 (4)	41 (7)
*Other results*			
DAS28	94 (16)	6 (1)	–
Radiological images	35 (6)	18 (3)	47 (8)
Interpretation of radiological images	38 (6)	31 (5)	31 (5)

^a^Percentages do not always add up to 100 % because of rounding

## Results

### Participants

Twenty-two care providers were interviewed in phase 1. All the nurses/nurse practitioners (*n* = 9) were female, with a mean age of 40 years (range 26–52). Among the interviewed rheumatologists (*n* = 13) five were male and eight were female, with a mean age of 46 years (range 38–64). All interviewees used the Internet regularly, also for work-related purposes. In phase 2, response on the questionnaire was 77 %, in total eight nurses/nurse practitioners and nine rheumatologists filled out the questionnaire, with a mean age of 44 years (range 28–64).

### Phase 1: interviews

#### Perceived advantages to patient home EMR access

Five main categories of advantages could be distinguished. First, a large majority of the interviewees felt that the service could improve patient participation and involvement in the treatment process. “I can imagine that as a patient, you’d like to look through your data before a consultation. That way, you’d be more prepared.” [Nurse practitioner, Female, 50 years old]. Second, several care providers mentioned that the service could enhance patients’ knowledge and make them better informed about their disease and treatment. This might enhance treatment adherence, since patients could have a better understanding of how and why their treatment works. Third, the service might affect the patient–provider interaction, due to the shared information and the openness of the record “People can have strange ideas on what doctors write about them. Sometimes there is a feeling of mistrust, which can be taken away by this.” [Rheumatologist, Female, 47 years old]. Furthermore, a few care providers reported that the service could positively influence patient safety, since the reading along of patients might reduce mistakes. “I think it forces us to write things down very precisely.” [Rheumatologist, Female, 43 years old]. Finally, improved communication with others was mentioned. Because patients would have their own data available, it could help them to recall what was said in the consult, which could enhance communication with relatives or other doctors. Also, patients would have their data available on vacation or abroad, which was perceived as useful in emergency situations.

#### Perceived disadvantages to patient home EMR access

Disadvantages of patient home access to the EMR could be divided into four categories. Many care providers assumed a lack of knowledge and understanding of EMR-data among a large amount of patients, which could cause fear and stress among patients due to misinterpretation. Second, the service could require extra work or a change in administration, for physicians might have to explain and clarify a lot more, both in consult and in the record itself. Third, some care providers feared that the EMR access would change the emphasis of the consultation and would absorb valuable consult time. “Then you get all sorts of questions if this or that is increased or decreased. Subsequently, you need 10 min to explain all these little things.” [Rheumatologist, Male, 61 years old]. Finally, the service could have a reverse effect on the patient–provider relationship, since a few care providers expected offended or worried patients due to physicians’ personal notes concerning a patient. “Sometimes you impetuously write your own conclusion of a consultation. For example, a comment about treatment adherence. You have to write that in the record somewhere, but it can be a sensitive topic.” [Rheumatologist, Female, 40 years old].

#### Preconditions for patient home EMR access

It is essential that the technology is solid and safe, to protect and ensure patients’ privacy. Furthermore, a number of care providers mentioned that the home-accessible EMR should be an extraction of the existing EMR, and not a separate record, to overcome extra work and mistakes. “It should not generate extra work, for example that you become obliged to type something about the lab results. Because then I’m just typing as a service towards the patient. And that is not what I need for myself, to pursue a good policy.” [Rheumatologist, Female, 44 years old]. The third precondition was a filter on the personal and clinical notes of the care provider. Most care providers felt it would be unpleasant and undesirable if patients read their clinical notes, which often contain jargon, personal considerations, and diagnostic considerations. “Sometimes you make a differential diagnosis, for we often have to think of cancer. While 99 % of the time it isn’t cancer, I do have to write it down. So, I wouldn’t let them read along in my clinical notes, it can be alarming. I’m not there to explain it” [Rheumatologist, Female, 49 years old]. Finally, opinions vary on the medical data that could be released to patients, including how and when this data should be presented. According to several care providers, information on lab results should not be available to patients until after the consult, in order to explain matters to patients, and to put the results into context. The results of phase 2 of our Delphi study present a complete overview of what medical data would be suitable to release to patients.

### Phase 2: questionnaire

Table 1 shows the answering frequencies of the items related to advantages of patient home access to the EMR. According to the respondents, the service could particularly have a positive influence on patients’ participation in treatment (65–88 %), their knowledge and self-management (77–82 %), and their communication with others (76 %). The most frequently agreed upon disadvantage, regarded patients’ skills to interpret their medical information (88 %) (Table 2). As for preconditions, according to 94 % of the respondents, the home-accessible record should be an extraction of the existing EMR, and not a separate record (Table 3). Furthermore, 82 % would prefer an inaccessible section in the record, to write down personal notes. A lag time on the release of lab data was an important precondition for 65 % of the respondents. Table 4 provides an overview of suitable content for a patient home-accessible EMR. Large parts of the disease and treatment data could be released to patients, according to most respondents. However, on more complicated outcomes, such as results of physical examinations, several lab results and radiological images, a differentiation is shown between the care providers. Moreover, most respondents (71 %) did not want patients to have access to their clinical notes.

## Discussion

Investigating rheumatology care providers’ opinions on patient home access to the EMR led to varying reactions. Expected positive consequences were an increase in patient participation in treatment, an increase in patient knowledge, self-management and patient safety, a more equal relationship between the patient and the care provider, and practical benefits in communication with others. Nevertheless, according to the respondents, patients might experience difficulties in interpreting medical data, which could cause (unnecessary) interpretation and communication problems, and an increase in workload. Overall, these findings agree with what is found in previous studies on care providers’ stands on this subject [5, 13, 19]. While the use of home-accessible EMRs in rheumatology practice is not described in literature yet, there are some examples from general clinical practice in which this service is adopted already. Generally, the key message confirms a number of positive outcomes, and very few negative ones [20–22]. It seems that the anticipated problem of worried or offended patients hardly occurs [3]. Several evaluations reveal that patients are generally enthusiastic about the access; they find it informative, they use it to prepare consults and they state that it enhances the understanding of their treatment [20, 21, 23, 24]. Positive outcomes concerning enhancement of trust in the care provider and more secure documentation are shown as well [3, 24, 25]. Earnest [10], furthermore, concluded that physicians in cardiology did not report any change in workload and no adverse consequences for their practicing procedures. Still, these former studies did reveal some necessary improvements. Patients regularly could not understand all the information in their record [8, 20] and they reported that they felt the need for aids to interpret (laboratory) tests or technical terms [10].

Our results on care providers’ views on preconditions and on suitable content to display to patients anticipate on these kind of difficulties. The results showed that most care providers do not want patients to view an overload of detailed information, in order to increase information provision without increasing confusion. Complex results as full blood test results and radiological images should not be available to patients, nor should providers’ clinical notes. Also, lab results should not be available to patients until after consult, in order to explain the information to the patient first. Thus, involving care providers in the first phases of the developmental process of patient services is essential.

This study is limited because of the small amount of respondents. However, we feel that the combination of interviews and a questionnaire, among care providers from different hospitals, has covered all main arguments. Further research on the implementation of a patient home-accessible EMR should focus on how care providers’ preconditions can be translated in the development of this service. A pilot study in clinical practice in which patients have access to their EMR is currently being set up, in order to study its’ advantages and disadvantages for all parties.

In conclusion, care providers are positive about providing patients home access to their medical record, because it might be a valuable next step into patient participation, patient empowerment, and in service towards the patient. In order to use this service in clinical practice, security must be optimal, and careful consideration of the content and presentation of the data is necessary. Participatory design, in which both patients and care providers are involved in the development of the service, is eminent to make this service useful in clinical practice.

## References

[1] Demiris G, Afrin LB, Speedie S (2008). Patient-centered applications: use of information technology to promote disease management and wellness. A white paper by the AMIA knowledge in motion working group. J Am Med Inform Assoc.

[2] Ross SE, Todd J, Moore LA (2005). Expectations of patients and physicians regarding patient-accessible medical records. J Med Internet Res.

[3] Ferreira A, Correia A, Silva A (2007). Why facilitate patient access to medical records. Med Care Compunetics.

[4] Honeyman A, Cox B, Fisher B (2005). Potential impacts of patient access to their electronic health records. Inform Prim Care.

[5] Van der Vaart R, Drossaert CHC, Taal E (2011). Patient preferences for a hospital based rheumatology Interactive Health Communication Application, and factors associated with these preferences. Rheumatology.

[6] Van der Vaart R, Drossaert CHC, Taal E et al (2010) Experiences and preferences of patients with rheumatic diseases regarding an interactive health communication application. In: Proceedings of the 2nd eTELEMED international conference on ehealth, telemedicine, and social medicine; 2010 Feb, Saint Martin 64–71 doi: 10.1109/eTELEMED.2010.16

[7] Richter JG, Becker A, Koch T (2010). Changing attitudes towards online electronic health records and online patient documentation in rheumatology outpatients. Clin Exp Rheumatol.

[8] Masys D, Baker D, Butros A (2002). Giving patients access to their medical records via the internet: the PCASSO experience. J Am Med Informs Assoc.

[9] Reti SR, Feldman HJ, Ross SE (2010). Improving personal health records for patient-centered care. J Am Med Inform Assoc.

[10] Earnest M, Ross SE, Wittevrongel L (2004). Use of a patient-accessible electronic medical record in a practice for congestive heart failure: patient and physician experiences. J Am Med Inform Assoc.

[11] Winkelman WJ, Leonard KJ (2004). Overcoming structural constraints to patient utilization of electronic medical records: a critical review and proposal for an evaluation framework. J Am Med Inform Assoc.

[12] Leonard K, Wiljer D, Casselman M (2008). An innovative information paradigm for consumers with chronic conditions: the value proposition. J Inf Tech Health Care.

[13] Dunn WN (1981). Public policy analysis.

[14] Adler M, Ziglio E (1996). Gazing into the oracle: the Delphi method and its application to social policy and public health.

[15] Linstone HA, Turoff M (1975). The Delphi method: techniques and applications.

[16] Members list of the Dutch Association for Rheumatology | [Ledenlijst Nederlandse Vereniging voor Reumatologie] (2010) Haarlem, DCHG Partner in medische communicatie

[17] Members list of the Dutch Health Professionals in Rheumatology | [Ledenlijst Nederlandse Health Professionals in de Reumatologie] (2010) Haarlem, DCHG Partner in medische communicatie

[18] Patton MQ (1990). Qualitative evaluation and research methods.

[19] Siteman E, Businger A, Gandhi T et al (2006) Clinicians recognize value of patient review of their electronic health record data. In: Proceedings of the AMIA symposium, Washington, DC, p 1101 PMC1839374 17238720

[20] Bhavnani V, Fisher B, Winfield M, Seed P (2010). How patients use access to their electronic GP record: a quantitative study. Fam Pract.

[21] Ross SE, Lin C (2003). The effects of promoting patient access to medical records: a review. J Am Med Assoc.

[22] Cardoso AB, Lima AF, Pereira AF et al (2005) The access to medical records: a systematic review. University of Porto, Faculty of Medicine [Internet]. Available from: http://medicina.med.up.pt/im/trabalhos05_06/sites/Turma2/files/final.pdf

[23] Ralston JD, Carrell D, Reid R (2007). Patient web services integrated with a shared medical record: patient use and satisfaction. J Am Med Inform Assoc.

[24] Fisher B, Bhavnani V, Winfield M (2009). How patients use access to their full health records: a qualitative study of patients in general practice. J R Soc Med.

[25] Weissman JS, Schneider EC, Weingart SN (2008). Comparing patient-reported hospital adverse events with medical record review: do patients know something that hospitals do not?. Ann Intern Med.

